# Effectiveness of intensive perioperative nutrition therapy among adults undergoing gastrointestinal and oncological surgery in a public hospital: study protocol for a pragmatic randomized control trial

**DOI:** 10.1186/s13063-022-06898-2

**Published:** 2022-11-26

**Authors:** A’ishah Zafirah Abdul A’zim, Zalina Abu Zaid, Barakatun Nisak Mohd Yusof, Mohd Faisal Jabar, Aainaa Syarfa Mohd Shahar

**Affiliations:** 1 Department of Dietetics, Faculty of Medicine and Health Sciences, Universiti Putra Malaysia, Serdang, Selangor Malaysia; 2 Department of Dietetics, Hospital Pengajar Universiti Putra Malaysia, Serdang, Selangor Malaysia; 3 Department of Surgery, Faculty of Medicine and Health Sciences, Universiti Putra Malaysia, Serdang, Selangor Malaysia

**Keywords:** Perioperative nutrition therapy, Early nutrition therapy, Surgical patients, Postoperative outcomes, Oral nutrition support

## Abstract

**Background:**

Perioperative malnutrition is common in patients undergoing gastrointestinal-oncology surgery and is associated with longer hospital stays, increased postoperative complications, poorer quality of life, and lower survival rates. Current practice emphasizes the role of early perioperative nutrition therapy as an early intervention to combat the postoperative complications of patients and the implementation is now widely adopted. However, there is still a lack of research on determining the effectiveness of intensive nutrition therapy and providing ONS perioperative locally. This becomes the significance of this study and serves as a basis for management and guideline in the local hospital settings.

**Methods:**

This is a pragmatic randomized control trial study where elective admitted patients will be randomly divided into the intervention (SS) or control (NN) group. All data will be collected during a face-to-face interview, anthropometric measurement, blood sampling (albumin, white blood count, hemoglobin, and c-reactive protein), handgrip strength, and postoperative complications. Group SS will be receiving a tailored lifestyle and intensively supplemented with oral nutrition support as compared to Group NN that will receive standard medical care. The primary outcome *for this study is* the length of stay in the hospital. Additional outcome measures are changes in biochemical profile and nutritional and functional status. The effects of intervention between groups on the outcome parameters will be analyzed by using the SPSS General Linear Model (GLM) for the repeated measure procedure.

**Discussion:**

The intervention implemented in this study will serve as baseline data in providing appropriate nutritional management in patients undergoing gastrointestinal and oncological surgery.

**Trial registration:**

ClinicalTrials.gov Protocol Registration and Results System (PRS) NCT04347772. Registered on 20 November 2019.

## Background

Recently, the definition of malnutrition, as proposed by the International Global Initiative on Malnutrition (GLIM), refers to a patient’s clinical condition that has at least one (1) phenotypical (involuntary weight loss, low body mass index, reduced muscle mass) or etiological criteria (reduced food intake or assimilation, inflammation, or disease burden) [1]. The levels of malnutrition severity are divided into Stage 1 (Moderate malnutrition) and Stage 2 (Severe malnutrition). This level can be determined by having one (1) phenotypic criterion that meets the grade [1].

Malnutrition is well reported in surgery patients [2, 3] especially those who have undergone major surgery [4–6], and particularly at risk in patients undergoing surgery for upper gastrointestinal or colorectal cancer [7, 8]. Preoperative malnutrition shows an increased length of hospital stay, a higher rate of surgical site infection and mortality [9], and is associated with higher postoperative complications, increased costs, poorer quality of life, and lower survival rates [9, 10]. Studies show a high prevalence of malnutrition or high nutritional risk during hospital admission [11, 12] but this is rarely assessed in the clinical setting, especially for patients who will undergo elective surgery.

The nutrition requirement for surgery is higher in comparison with the normal requirement to support a speedy recovery. Suboptimal nutritional intake will cause further depletion of the patient’s nutritional status preoperatively and a higher risk of postoperative complications. Interestingly, one of the Malaysian studies shows a significant number of post-surgery complications compared to pre-surgery which was associated with a poor level of nutrition [10].

Nutrition therapy is the provision of nutrition or nutrients either orally (regular diet, therapeutic diet, e.g., fortified food, oral nutritional supplements) or via enteral nutrition (EN) or parenteral nutrition (PN) to prevent or treat malnutrition [13] which includes dietary counseling. Nutritional therapy is important as it affects mainly the perioperative maintenance of the nutritional state to prevent postoperative complications [14]. It is strongly recommended not to wait until severe disease-related malnutrition has developed, but to start nutrition therapy early, as soon as a nutritional risk becomes apparent [15].

In addition, for patients with severe nutritional risk in surgical patients with oral feeding, some improvements were shown, resulting in shortened duration of hospital stay and flatus, and some wound and infectious complications [16]. Current practice emphasizes the role of early nutrition therapy as an early intervention to combat the postoperative complications of patients and the implementation is now widely adopted. However, there is minimal local data to present the effects of perioperative nutrition therapy and the process of nutrition management, from screening/assessment on admission to nutritional support and monitoring for surgical patients. Therefore, there is a need for a study in this area to find good practices that should be mandated in routine patient care regardless of disease type, specifically in a local hospital setting.

### Primary objective

The primary objective is to determine the effect of intensive perioperative nutrition therapy on *length of hospital stay* in adult patients undergoing major elective gastrointestinal and oncological surgery over an extended period before hospital admission, during the hospital stay, and after discharge in comparison to a control group of the same type of patients who will receive the standard care.

## Methods/design

### Design

This study will be a single-blinded, pragmatic randomized clinical trial with two arms comparing an intervention group with a control group involving sixty-eight adult patients undergoing major elective gastrointestinal and oncological surgery in Hospital Serdang (HSD).

**Fig. 1:**
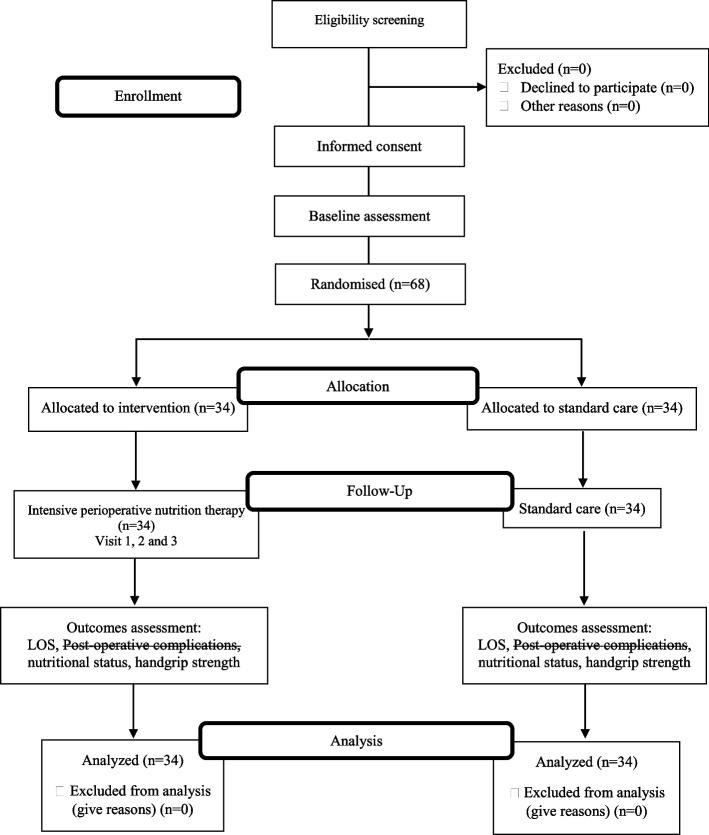
Flow diagram

**Fig. 2:**
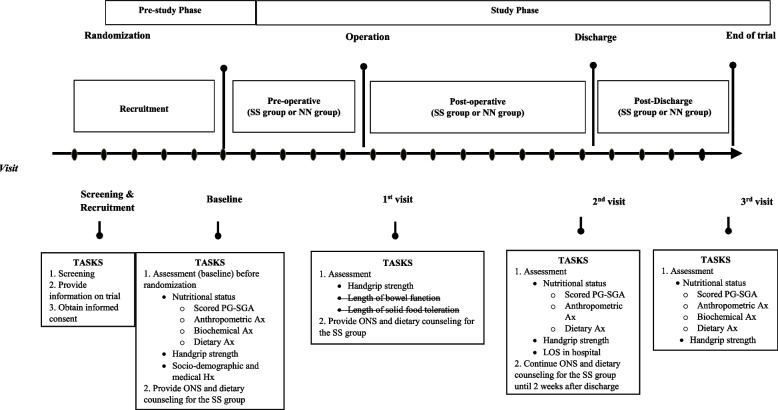
A graphical presentation of the protocol and timeline of the intervention study. Ax: assessment; Hx: history; PG-SGA: Patient-Generated Subjective Global Assessment; ONS: oral nutrition support; LOS: length of stay; SS: intervention group; NN: control group

A pragmatic trial is a randomized controlled trial whose purpose is to inform decisions and the benefit of treatment in clinical practice [17]. Critical aspects of pragmatic trials are that they are conducted in the standard practice setting, they include typical participants to whom the intervention will be applied in practice, the intervention is flexible as it would be in practice, and a range of directly relevant outcomes are assessed [17, 18]. Pragmatic trials indicate the success or failure of realistic interventions in a clinical setting [19]. The RCT conformed to the Consolidated Standards of Reporting Trials (CONSORT) statement for reporting RCT with two arms comparing an intervention group with a control group [20]. After consenting to participate, subjects will be allocated into intervention (SS) and control (NN) groups randomly. The flow diagram for recruitment and randomization is shown in Fig. 1. A graphical presentation of the proposed study procedure and timeline is shown in Fig. 2. The schedule of enrolment, interventions, and assessments are in Table 1 [21].

**Table 1:**
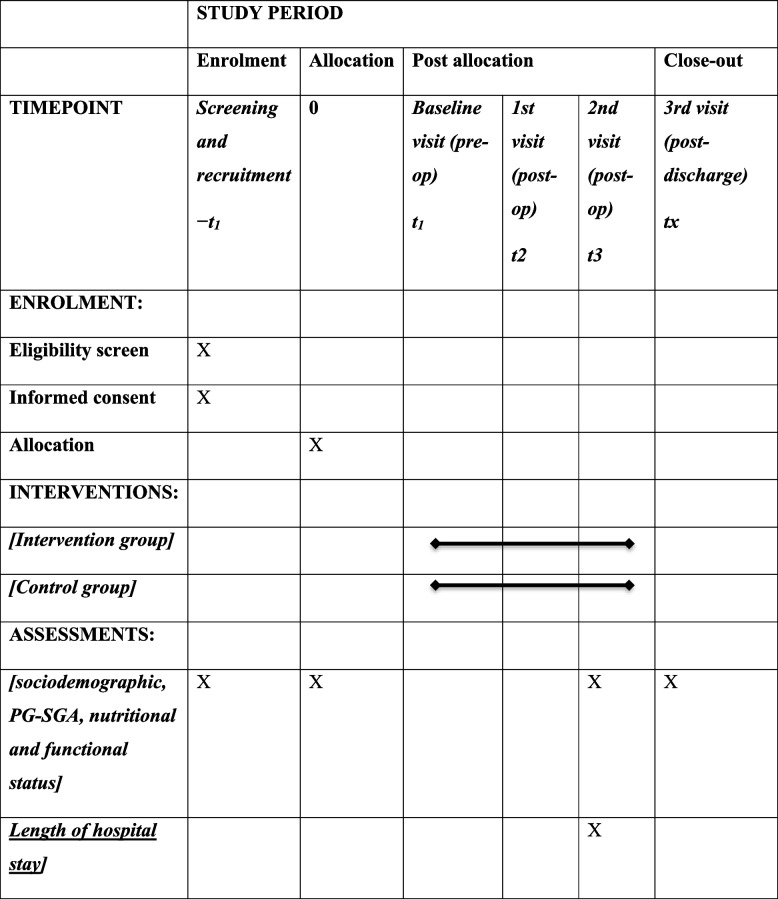
The schedule of enrolment, interventions, and assessments

*−t*_*1*_*:* Outpatient appointment day

*t*_*1*_*:* First day of hospital admission for planned surgery

*t*_*2*_*:* First day of tolerating solid food in the ward

*t*_*3*_*:* 1 day before discharge from hospital

*t*_*x*_*:* 4 weeks after discharge from hospital

### Study population

Men and women who plan for surgical procedures will be invited to participate in this study with informed consent. A screening form will be used to exclude those who do not meet the inclusion criteria. The inclusion criteria are those who are receiving elective major surgery treatments for gastrointestinal and oncology-related surgery, aged from 18 to 65 years old, Malaysian, able to communicate verbally, Malnutrition Screening Tool (MST) score ≥ 2, and provided and signed informed consent. The exclusion criteria are those who are receiving elective major surgery treatments other than gastrointestinal and oncology-related including bariatric surgery, those who have received preoperative enteral or PN, those who require emergency surgery, complicated with chronic diseases and fluid retention (renal/ cardiovascular/pulmonary/ hepatic), and those who have participated in other research studies.

### Recruitment

Potential patients who are attending the surgical clinic will be approached by the project team member. They will inform all potential participants of the study procedures. However, the study is single-blinded when the investigator but not the study participants knows which treatment has been allocated. The patient information sheet and consent form will be given to the eligible subjects who have agreed to be recruited for the study. Subjects will be allowed to bring back the consent form if they are keen to seek advice from their family member. The consented participants will be randomized into one of the two groups; either into intervention (SS) or control (NN) group.

### Randomization

An online platform of randomization method will be used to prevent risk allocation concealment. The permuted block randomization will be performed using the following platform, i.e., https://www.sealedenvelope.com [22]. The randomization will be based on a block method for a block size of four, with A for the intervention group and B for the control group. This block of four has six different possible arrangements of two As and Bs. The randomization with body mass index (BMI) and gender stratification will be conducted to assign participants to either Group SS: intervention group or Group NN: control group. The flow diagram for recruitment and randomization is shown in Fig. 1.

### Study procedure

All data will be collected during a face-to-face interview, blood sampling, and direct anthropometric measurement with the participants at Hospital Serdang starting from before operation until 2 weeks after discharge.

#### Intervention group, Group SS

Group SS will be receiving tailored, more intensive, and ongoing nutrition support and lifestyle advice as compared to Group NN. They will be supplemented with oral nutrition support (ONS), available in 400g/can, providing 226 kcal/serving and 9.6 g protein/serving. ONS will be prescribed according to the patient’s requirement, from 1 to 4 servings a day*.* ONS consumption should be daily from 7 to 14 days until 6 h before the scheduled surgery. Patients are encouraged to consume ONS in a small portion, frequently and in between meals. They also will receive intensive and detailed nutrition counseling at each visit. Nutrition counseling consists of adequate energy and protein according to requirement, fluid and salt intake, replenishment of micronutrients, and controlling blood glucose levels before surgery. Follow-up phone calls will be done from time to time to monitor their progress.

#### Control group, Group NN

Participants in Group NN which is referred to as the control group will receive the standard care of the clinic without getting supplemented with ONS. Nutritional advice will be based on a guideline specifically focused on the treatment of symptoms such as nausea, vomiting, loss of appetite, and diarrhea, and how to deal with the symptoms through nutritional approaches. The advice will be given by clinicians or nurses in the clinic. Those who are identified as having moderate and severe malnutrition will be referred to a dietitian for nutrition counseling. Participants in this group will also have to follow up through phone calls from time to time to assess and monitor their nutritional status before a scheduled surgery. The researcher will attend to any problems faced by participants in this study.

All participants will receive standard postoperative care from clinical and nurse staff with the commencement of free fluids and reintroduction of the normal diet without interference from the researcher or protocol. ONS will be given after surgery, only to the intervention group (Group SS) starting on the first day of nourishing fluid/diet allowance until the day of hospital discharge according to the prescription given. On the other hand, participants in the Group NN would not be given any ONS at postoperatively (Table 2).

**Table 2:** ONS allocation

Group	Preoperation	Postoperation
Group 1 (SS)	Supplements	Supplements
Group 2 (NN)	No supplements	No supplements

All participants will have to follow up either at an outpatient clinic or home visit 4 weeks after hospital discharge. Regular (fortnightly) contact with all participants will be maintained for 4 weeks after discharge to monitor their nutritional status and progress. Control groups will be treated exactly as the intervention group apart from the ONS provided.

Participants also will be monitored for complications throughout the study period. Monitoring will occur by daily observation of the participant, checking their medical and nursing notes, and liaising with the attending surgical team and nursing staff. At the same time, complications will be noted as major or minor by using a validated criterion [23].

After the trial ends, participants are encouraged to contact the research team via telephone or email should any issues or concerns arise. Participants will be informed if their assessment results are outside the acceptable or normal range and will be encouraged to seek medical advice. Participants are encouraged to continue physician’s treatment and follow-up after surgery, and if needed, the researcher will help those who are at nutritional risk to seek advice from a dietitian as a continuation of care, regardless of which group (intervention or control group).

### Criteria for withdrawal

The participation will be voluntary, and the participants are free to withdraw from the study at any time any justification and this will in no way affect their future treatment.

Other possible criteria for drop-out/withdrawal are if the participant develops adverse events (AEs), suddenly has abnormal laboratory values, participants develop a condition during the study that violates the inclusion/exclusion criteria, the participant did not proceed to a qualifying operation, enteral and/or parenteral nutrition is administered after surgery, prolong fasting/nil by mouth (NBM) due to any procedure, and particular prescription from a physician that will interfere with the intervention.

### Data collection procedure

Each participant will be assessed in the clinic/wards during four different sessions (baseline, first, second, and third visits) over the study period (Fig. 2). During the baseline visit, screening and recruitment will be conducted at the surgical clinic, attended by potential participants who have appointments for examination and are in line to get surgery appointments. Potential participants are those who are scheduled to have surgery and meet the inclusion and exclusion criteria. The scheduled project team member will be assigned to approach and inform all potential participants about the study procedures. The patient information sheet and consent form will be given to eligible subjects who have agreed to be recruited for the study. Subjects are allowed to bring back the consent form if they are keen to seek advice from their family members. Agreed and consented participants will be randomly sampled and divided into two (2) groups.

During the baseline visit (pre-study phase), the researcher will be obtaining the patients’ history of dietary intake via 24-h dietary recall. Based on this assessment, the researcher will be able to determine participants’ estimated intake based on their estimated requirements. The dietary advice will be given individually and is structured based on ESPEN guidelines for surgical patients [15].

Participants who are allocated to Group SS will be supplied with ONS, a maximum of 4 servings/day according to their requirements. Participants need to consume the supplied ONS starting from recruitment until 6 h before surgery. Follow-up through phone calls from time to time will be done to assess participants’ compliance with the supplied ONS. During the study phase, participants need to consume ONS once allowed, and compliance will be assessed daily until discharge. Participants will be asked to keep the ONS packaging material during both phases. The researcher will collect the packaging material during the 1st visit and the day of hospital discharge. While Group NN will not be supplemented by any of ONS in the pre-study phase and study phase.

During visits 1, and visit 3, dietary intake data will be collected using a self-administered 2-day 24-h dietary record. All the participants will also be given comprehensive verbal instructions on the method of recording food intake using the 2-day 24-h dietary record. The illiterate participants will be asked to seek assistance from their family members to record for them. They will be asked to complete the 2-day 24-h dietary record of food intake before their next visit, which will be used as the basis for their individualized dietary advice.

During the baseline visit, all data collection will be conducted including a socio-demographic questionnaire, with exception of data for *length of hospital stay* which can only be obtained at visit 2. Meanwhile, anthropometric measurements (except height) and PG-SGA questionnaire, the same methods and instruments will be used repeatedly at visit 1 and visit 3. Data for handgrip will be conducted at every visit while biochemical data will be collected for a second time at the end of the study which is during visit 3. The details of the assessment and procedure are shown in Table 3.

**Table 3:** The assessment procedure at (baseline), visit 1, visit 2, and visit 3

The assessment procedure	Visit			
	Baseline	1	2	3
**Questionnaire**				
Social demographic background	/			
The scored PG-SGA	/		/	/
**Dietary assessment**				
1-day 24-h dietary recall	/			
2-day 24-h dietary record			/	/
**Anthropometric measurements**				
Body weight	/		/	/
Height	/			
Mid-arm circumference	/		/	/
Triceps skinfold thickness	/		/	/
**Biochemical data**				
Serum albumin—Medical report	/			/
White blood count—Medical report	/			/
Hemoglobin—Medical report	/			/
C-reactive protein	/			/
**Functional status**				
Handgrip strength	/	/	/	/
**Complications postoperative**				
Length of stay in hospital			/	

### Primary outcome measure

#### Length of hospital stay

Length of hospital stay will be recorded starting from postoperative day 1 until the day of the patient’s discharge. The total length of hospital stay will be taken during visit 2, the day of the patient’s discharge.

### Secondary outcome measures

#### Nutritional status

##### The scored Patient-Generated Subjective Global Assessment (PG-SGA©)

The scored PG-SGA© consists of two sections with seven domains. The first section, which will be completed by the participant, is in the form of a checklist and comprised weight, food intake, symptoms, activities, and function. The second section will be completed by the researcher as it covers the disease and its relation to nutritional requirements, and determination of metabolic demands, and will be followed by a nutrition-related physical examination, including subjective body composition (i.e., fat, muscle, and fluid status).

A score ranging from 0 to 4 will be given for each domain depending on the impact on the nutritional status. PG-SGA© scores that range from 0 to 35 reflect a greater risk of malnutrition or indicate a lower nutritional status of the patient. These scores will be transformed into global ratings—Stage A, Stage B, and Stage C—which will represent the states of being well-nourished, moderately malnourished, and severely malnourished, respectively. Then, the criticality of the need for nutritional intervention will be identified and classified based on the scores with 0 to 1 point for those requiring no intervention, health education for 2 to 3 points, dietetic intervention for 4 to 8 points, and nutrition support for ≥9 points.

##### Anthropometric measurements

The anthropometric measurements will be carried out in this study including body weight, height, mid-arm circumference (MAC), and triceps skinfold thickness (TSF). These measurements will be taken directly before the interview with all the participants.

Body weight and height measurements will be taken while participants are barefooted and wearing lightweight clothing with empty pockets, without watches, or other accessories. Weight will be determined to the nearest 0.1 kg using a digital weighing scale (SECA, British Indicators Ltd., UK). The machine will be calibrated every morning with a standard weight before it is used. Height will be measured in the standing position to the nearest 0.1 cm using a SECA 206 microtoise tape (Vogel and Halke GmbH & Co, Hamburg, Germany) which will be attached to the wall. The participant will be asked to stand straight with their head in the Frankfort plane, feet together, knees straight, and heels, buttocks, and shoulder blades in contact with the vertical surface of the wall.

MAC and TSF will be taken without any sleeve on the measuring arm, watches, or other accessories. The subject will be in a relaxed standing position with the arms hanging by the sides. MAC will be measured in the middle arm (same distance) from acromiole and radiale bone, to the nearest 0.1 cm using SECA 201 circumference measuring tape (SECA, British Indicators Ltd., UK). Meanwhile, TSF will be measured at the most posterior part of the triceps when viewed from the side at MAC level, to the nearest 0.1 mm using a Harpenden skinfold caliper (HaB International Ltd, UK). The caliper will be calibrated every morning before it is used to minimize measurement errors. All anthropometric measurements will be taken twice by the same investigators, and the average will be used. The weight and height of the participants will then be used to calculate the BMI [24].

##### Biochemical data

Data on serum albumin, white blood count (WBC), and hemoglobin level will be obtained from the patient’s medical report at baseline and at the end of the visit. For other blood parameters, 10 ml of venous blood will be taken following an overnight fast. The samples will be separated immediately using centrifugation (1800*g* for 10 min at 4°C). The serum will be stored at 80°C until analysis for the measurement of C-reactive protein, which will be performed after all samples are collected.

##### Dietary intake

Dietary intake will be measured through a 24-h dietary recall at baseline and 2 days of 24-h dietary records during visits 2 and 3. The participants will be required to keep a record of all food and beverage intakes within every 24 h of data collection. The participant will be asked about their intake before the visit. Details of food information and descriptions, which include brand names, preparation, and cooking methods, as well as recipes of any mixed dishes eaten during the study period, will also need to be recorded. In situations where any foods and beverages are consumed away from home, the participants will be encouraged to describe the quantities consumed using the household measurements as well (e.g., glass, cup, Chinese rice bowl, plate, tablespoon, and teaspoon).

The researcher will explain to both groups how to record their 2-day intake in a food diary. They will also be given a detailed set of instructions together with a food album. The food album lists commonly consumed food and includes details of portion sizes relative to typical household measurements to facilitate recalls of serving size and improved accuracy.

A computerized local dietary analysis program, Nutritionist Pro version 2.0 (First Data Bank, The Hearst Corp. USA) will be used to analyze the nutrient intakes of the patients. The foods and beverages consumed by the participants will be coded by type and amount which then will be analyzed for nutrient content primarily based on the Malaysian Food Composition Database [25]. Nutritionist Pro will then be used to calculate dietary intake at baseline (based on one 24-h dietary recall), and at other subsequent assessment time points (based on average intake over every 2 days of dietary record).

##### Handgrip strength

Handgrip strength is measured on the non-dominant hand using a Jamar hand dynamometer (Fred Sammons Inc, Burr Ridge, Illinois, USA). Handgrip strength as a surrogate marker for muscle strength in patients with cancer has been well documented elsewhere. Subjects will be sitting with their shoulder adducted and neutrally rotated, elbow flexed at 90°, forearm in the neutral position, and standard verbal instructions will be given to the subjects to squeeze the dynamometer as hard as possible for three times after an interval of 5 s in between grips. An average of three successive attempts will be used as the final result.

### Other variables

#### Participant details and demographics

Information on the participants’ address, contact number, date of birth, age, sex, ethnic group, education level, job status, income level, and marital status will be recorded.

#### Medical history

Investigation of participants’ medical history on the type of disease, duration, treatment obtained, and medication received.

### Statistical analysis

#### Sample size calculation

A systemic review study in 2016 showed that early feeding has a significantly shorter length of hospital stay (*p*<0.01) compared to the late timing of feeding with a difference of −1.72 days and not associated with an increase in clinically relevant complications [26]. Several other studies also support early nutrition for surgery patients significantly shortening the length of hospital stay [27–29].

LOS was chosen to calculate the sample size because it has been used widely as a primary outcome in most nutrition-related RCT studies [30–32] among patients undergoing surgery. The sample size was calculated based on the data from a study conducted on colorectal cancer patients undergoing surgery [30]. The primary outcome was calculated based on the difference in LOS between two groups (control and intervention group) which was 3.45 days.

A large effect size of 0.8 for clinical significance was assumed, and practicality for this research was considered, with a power (*β*) of 90% and a probability (*α*) of 0.05. Allowing for a “drop-out” rate of 20% for the RCT study, we aim to recruit participants who will be included in each group, with the final sample size calculated as 68 participants with 34 participants in each group, respectively.

### Analysis of results

The data collected will be analyzed by using the statistical software Statistical Package for Social Sciences (SPSS) and checked for normality via Kolmogorov-Smirnov analysis. All data will be normally distributed as indicated by *p*>0.05 unless otherwise stated. If the data will be not normally distributed, analyses will be carried out on the natural logarithm of the values to improve the symmetry and homoscedasticity of the distribution.

Descriptive statistics, including percentages, mean values, and standard deviations, will be used to describe the baseline demographic data, type of diseases, nutritional status level including anthropometric data, and dietary intake. The median will be used when the data are skewed. When the data become skewed, the mean loses its ability to provide the best central location for the data because the skewed data are dragging it away from the typical values. In addition, the median is not strongly influenced by skewed values.

The values from both groups will be compared using an ANOVA test for approximately normally distributed data, followed by tests on pairs of groups using the Bonferroni adjustment. Non-normal data will be compared by the Kruskal-Wallis test followed by the Mann-Whitney *U* test. ANCOVA or logistic regression will be used with models adjusted according to potential confounders to determine predictors of changes in the key outcome variables of the length of hospital stay with *p*<0.05 significance level. An intent-to-treat (ITT) analysis will be performed to determine the effect of the intervention study on the assumption that participants have adhered to the dietary advice and ONS at the start of the intervention study and had baseline and endpoint values. In other words, ITT analysis includes every randomized subject; according to randomized treatment assignment regardless of their non-compliance, subsequent withdrawal from treatment, or deviation from the protocol and anything that happens after randomization. The effect of intervention between treatment groups on outcome parameters will be carried out by using General Linear Model (GLM) for repeated measure procedure. This method is quite robust to violation of the assumption of normality [33].

A partial Eta-squared measure will be used to compare the magnitude of changes with the large effect size, indicating a significant difference in magnitude [34]. Using Cohen’s classification, the value of partial Eta-squared is considered to be a small effect size at 0.01, a moderate effect size at 0.06, and a large effect size at 0.14 [35].

## Discussion

Globally, hospital malnutrition is under-recognized, under-diagnosed, and under-treated by health care professionals, which may potentially be the cause of the omission of nutritional assessments in routine patient assessments on admission [36]. The standardized process of nutrition assessment will be measured for both the intervention and control groups in the study which will be used in routine patient care.

The provision of early nutrition intervention or perioperative enteral nutrition in surgical patients is intended to optimize the patient’s nutritional status, prevention, and treatment of catabolism, lower the incidence of minor complications during surgery, and be cost-effective [31]. In addition, appropriate perioperative nutrition therapy has shown improvements in perioperative outcomes, especially in gastrointestinal and oncologic surgical patients [3].

Moreover, most studies have investigated the effect of nutrition therapy on postoperative complications in which ONS was prescribed during the preoperative but not after the surgery even though postoperative ONS also has been shown to have beneficial effects on the outcome. Intensive perioperative nutrition therapy is aimed to overcome the pre- and post-surgical complications because its efficacy in improving the nutritional status of the participants has been observed.

In parallel, the use of ONS, together with voluntary food intake, as a means of providing nutritional support to surgical patients is more straightforward, easy to administer, comparatively cheap, free from complications, and palatable given the range of flavors available [31, 32]. Intensive perioperative nutrition therapy will be a good choice to practice among dietitians in clinical settings to provide a good outcome for surgical patients.

### Trial status

The protocol version number is NCT04347772 and the date of registration is 20 November 2019. The date of recruitment started on 31 August 2021 and was completed on 31 December 2021.

## Data Availability

The datasets used and/or analyzed during the current study are available from the corresponding author upon reasonable request.

## Abbreviations

EN: Enteral nutrition
PN: Parenteral nutrition
RCT: Randomized control trial
CONSORT: Consolidated Standards of Reporting Trials
SS: Intervention group
NN: Control group
MST: Malnutrition Screening Tool
BMI: Body mass index
ONS: Oral nutrition support
ESPEN: The European Society for Clinical Nutrition and Metabolism
LOS: Length of stay
PG-SGA©: Patient-Generated Subjective Global Assessment
MAC: Mid-arm circumference
TSF: Triceps skinfold thickness
WBC: White blood count
*β*: Power
*α*: Probability
SPSS: Statistical Package for Social Sciences
MREC: Malaysia Research and Ethics Committee
GLIM: Global Initiative on Malnutrition
ITT: Intent-to-treat
GLM: General Linear Model
AEs: Adverse event/s
NBM: Nil by mouth
